# Prevalence of increased transaminases and its association with sex, age, and metabolic parameters in children and adolescents with obesity – a nationwide cross-sectional cohort study

**DOI:** 10.1186/s12887-021-02747-4

**Published:** 2021-06-09

**Authors:** Resthie R. Putri, Thomas Casswall, Emilia Hagman

**Affiliations:** Division of Pediatrics, Department of Clinical Science, Intervention and Technology, Karolinska Institutet, Blickagången 6A, 141 57 Huddinge, Stockholm, Sweden

**Keywords:** Pediatric obesity, Non-alcoholic fatty liver disease, Fatty liver, Alanine aminotransferase, Epidemiology

## Abstract

**Background:**

Childhood obesity increases the risk of non-alcoholic fatty liver disease marked by elevated alanine aminotransferase (ALT). This study investigated the prevalence of increased ALT in children and adolescents with obesity, and its associations with sex, age, degree of obesity, and metabolic parameters.

**Methods:**

Individuals between 5 and 17.99 years of age enrolled in the Swedish Childhood Obesity Treatment Register (BORIS) before March 2020 were included. Mildly increased ALT was defined by ALT 27–51 U/L (males) and 23–43 U/L (females), while markedly increased ALT by levels above. Multiple logistic regression models were used for statistical analysis.

**Results:**

Among 11,776 individuals (age 11.0 ± 3.3 years, 53.5% males), the prevalence of mildly and markedly increased ALT were 37.9 and 10.6%, respectively. A sex-age interaction was found, where increasing age strengthened the odds of markedly increased ALT in males (OR, 99% CI: 1.34, 1.29–1.4 for each year) while the corresponding pattern in females with was minuscule (1.09, 1.02–1.10). Compared to class I obesity, class II and III obesity had greater odds ratios for mildly increased ALT (class II obesity OR, 99% CI: 1.51, 1.35–1.70; class III obesity OR, 99% CI: 2.17, 1.66–2.61) and for markedly increased ALT (class II obesity OR, 99% CI: 1.82, 1.51–2.20; class III obesity OR, 99% CI 3.38, 2.71–4.23). Dyslipidemia was associated with both mildly and markedly increased ALT, all *p* < 0.001. Prevalence of impaired fasting glucose was 19.1% in normal ALT group, 20.4% in mildly increased ALT group, and 29.0% in markedly increased ALT group.

**Conclusions:**

The risk of markedly increased ALT increased exponentially with age among boys, but not among girls. Higher degree of obesity was observed in individuals with mildly and markedly increased ALT. Further, metabolic derangements were more prevalent among individuals with mildly and markedly increased ALT.

## Introduction

The worldwide prevalence of obesity among children and adolescents has increased dramatically in the past four decades [1]. The consequences of pediatric obesity are evident as it is associated with both impaired metabolic health [2–4], psychosocial boundaries [5, 6], and premature mortality [7]. The obesity epidemic has also contributed to the rise in pediatric non-alcoholic fatty liver disease (NAFLD) [8, 9]. Pediatric NAFLD is defined as accumulation of 5% or more hepatocytes with fatty infiltration, in the absence of excessive alcohol consumption or other causes of liver disease [8]. Pediatric NAFLD has become the most common chronic liver disease, and may progress to fibrosis and cirrhosis [8, 10]. Furthermore, children and adolescents with NAFLD are more likely to have impaired glucose metabolism [11] and vascular changes [12] compared with those without NAFLD.

Although childhood obesity is well known to be closely associated with NAFLD [8, 13, 14], pediatric obesity and NAFLD, especially in young children, are not concomitant [9, 14]. The majority of children and adolescents with obesity do not have NAFLD [13, 14]. Among children and adolescent with obesity, males and adolescent age have been reported to be independent risk factors of NAFLD [11, 15, 16]. However, the presence of interaction effect between sex and age on pediatric NAFLD has not been investigated, whereas this interaction has been known to modulate NAFLD risk in adults with obesity [17, 18]. Examining this sex-age interaction is essential as it may contribute to the better understanding of pediatric NAFLD pathogenesis and pathophysiology.

In addition, previous studies assessing the association of pediatric NAFLD with impaired fasting glucose and dyslipidemia have demonstrated inconsistent results. A population-based and some hospital-based studies in children and adolescents with obesity found associations of NAFLD with impaired fasting glucose [19–21] and all dyslipidemia components [22], yet other studies detected neither association of NAFLD with impaired fasting glucose [15] nor with all dyslipidemia components [21, 23]. Identifying which children and adolescents with obesity that is having the greatest risk of NAFLD is important since early detection and prompt treatment of NAFLD in this high-risk population can be effective to prevent progression and reverse the disease [8].

The gold standard to diagnose pediatric NAFLD is liver biopsy [14, 24]. However, its feasibility and usefulness in children and adolescents are limited since liver biopsy in children is recommended to be performed using general anesthesia, has several possible severe complications, is costly and is not widely accessible [8, 9, 24]. The liver enzyme serum alanine aminotransferase (ALT) is commonly used as a surrogate marker of suspected NAFLD and is considered as the best screening tool of pediatric NAFLD up to now [8, 20]. This study aimed to investigate the prevalence of varying degrees of increased ALT in a large cohort of children and adolescents with obesity and to assess the associations of increased ALT with sex, age, degree of obesity, and metabolic risk parameters.

## Methods

### Study population

We conducted a cross-sectional study from a prospective cohort of children and adolescents registered in the Swedish Childhood Obesity Treatment Register (BORIS). BORIS is a register for long-term monitoring of obesity treatment in children and adolescents. The obesity treatment consisted mainly of behavioural lifestyle modifications. Data of anthropometrical measurements were collected in each clinical visit. Laboratory analyses of blood samples, such as ALT, glucose, and fasting lipid profile (triglycerides, cholesterol, HDL, and LDL) were measured as determined by the clinical needs and current national guidelines. Details about BORIS are published elsewhere [25]. In Sweden, all health care for children and adolescents up to 18 years, including obesity treatment, is free of charge.

**Fig. 1:**
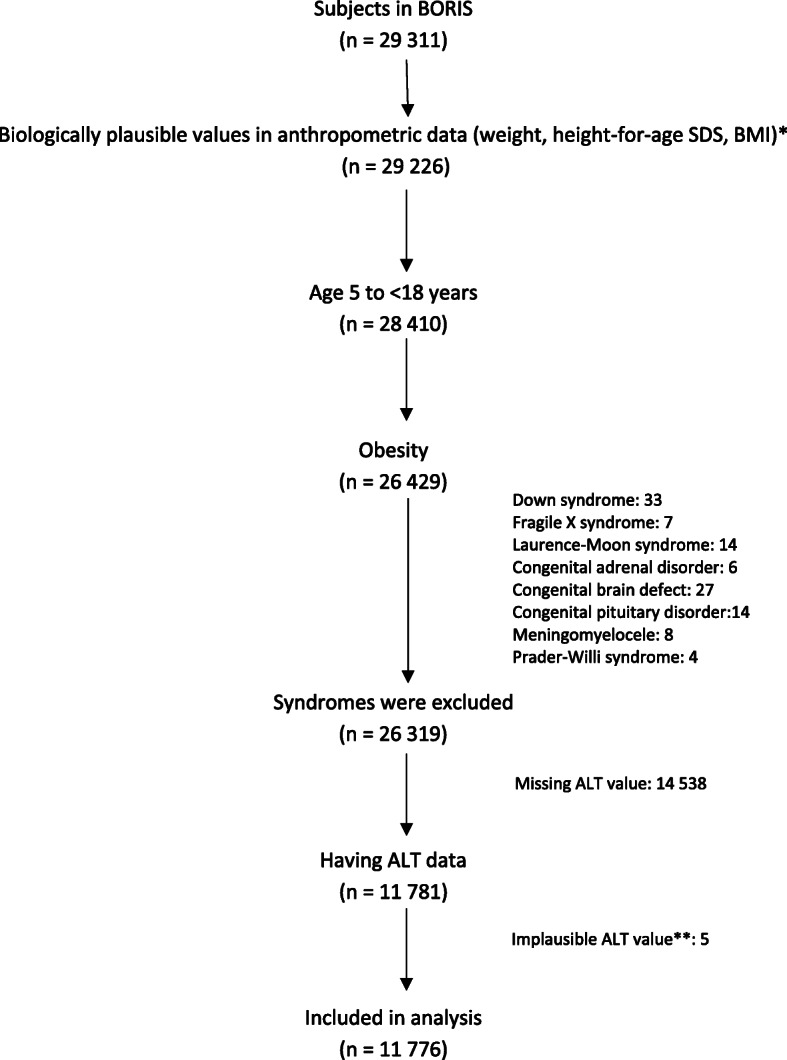
Flowchart of the exclusion process of the cross-sectional study. Abbreviations: BMI, body mass index; SDS, standard deviation score; ALT, alanine aminotransferase. * Weight ≤ 200 kg, BMI < 80 kg/m2, height-for-age z-score is between − 5 and 5. ** ALT ≥361 U/L

Inclusion criteria were individuals between 5 and 17.99 years of age enrolled in BORIS between December 1, 1994, and February 29, 2020, who had obesity according to the International Obesity Task Force [26], and had ALT-data. Exclusion criteria were presence of syndromes or congenital abnormalities that might interfere with obesity and/or serum ALT level, i.e. Down syndrome, Fragile X syndrome, Laurence-Moon syndrome, Prader-Willi syndrome, congenital brain defect, congenital pituitary disorder, and meningomyelocele. Other causes of increased transaminases in children (such as viral hepatitis and Wilson’s disease) could not be excluded due to missing data. After the exclusion process, 11,776 individuals were included in the analysis. The flowchart is shown in Fig. 1.

Patients and guardians are informed about the register, and approval of registration is documented in the patient’s electronic medical file. An opt-out approval is the current law and regulation in Sweden. The study was approved by the Regional Ethical Review Board in Stockholm (number 2014/381–31/5).

### Measurements

The outcome was the first registered ALT measurement. Elevated ALT has long been used as a measure of liver inflammation and is considered as a reliable marker for NAFLD in the pediatric population [27]. Most cases of pediatric NAFLD have mild elevation of ALT [28], yet mild elevation of ALT may be caused by other conditions than NALFD, such as viral infections, muscle disease, and drug-induced liver injury [29]. Nevertheless, a high level of ALT (i.e. two times upper limit of normal reference level) in children and adolescents with obesity is most likely due to NAFLD [8]. We, therefore, divided the outcome into *mildly increased* ALT and *markedly increased* ALT. As the reference values for ALT varies markedly according to different laboratories in Sweden, we chosed to use the sex-specific reference values for children which have been suggested by NASPGHAN [8]. Thus, the upper reference level (URL) was defined as 22 U/L (0.37 μkat/L) for females and 26 U/L in males (0.44 μkat/L) [8]. Serum ALT level was classified as “normal ALT” (≤ URL), “mildly increased ALT” (> URL to < 2 times URL), and “markedly increased ALT” (≥2 times URL) [8].

The primary exposure variables were: age, sex, and degree of obesity, which were measured within 2 weeks before or after ALT. As body mass index (BMI) varies with age and sex in growing children, body mass index standardized age- and sex-dependent deviation scores (BMI SDS) was used to measure the degree of obesity in children and adolescents [26]. We classified the individuals into class I obesity, class II obesity, and class III obesity based on age- and sex-specific curves corresponding to adult BMI of 30, 35, and 40 kg/m^2^, respectively [26, 30].

Other exposure variables were the presence of impaired fasting glucose and dyslipidaemia, recorded simultaneously with ALT. Impaired fasting glucose was defined according to the World Health Organization as fasting glucose ≥6.1 mmol/L [31]. Lipid profile was classified as the following: total cholesterol “acceptable” (< 4.4 mmol/L), “borderline-high” (4.4 to 5.1 mmol/L), “high” (≥5.2 mmol/L); low density lipoprotein cholesterol (LDL): “acceptable” (< 2.8 mmol/L), “borderline-high” (2.8 to 3.3 mmol/L), “high” (≥3.4 mmol/L); high density lipoprotein cholesterol (HDL): “low” (< 1.0 mmol/L) and “acceptable” (≥1.0 mmol/L), triglycerides: “acceptable” (< 0.8 mmol/L in individuals aged 5 to 9 years, or < 1.0 mmol/L in individuals aged 10 to 18 years), “borderline-high” (0.8 to 1.1 mmol/L in individuals aged 5 to 9 years, or 1.0 to 1.5 mmol/L in individuals aged 10 to 18 years), “high” (≥1.2 mmol/L in individuals aged 5 to 9 years, or (≥1.6 mmol/L in individuals aged 10 to 18 years) [32]. In individuals who had fasting glucose and fasting insulin data, homeostasis model (HOMA) for insulin resistance was calculated. HOMA was computed with the formula: fasting plasma glucose (mmol/L) times fasting serum insulin (μU/mL) divided by 22.5. All the blood analyses were performed by accredited laboratories in Sweden. All methods in the study were carried out in accordance with the Helsinki guidelines and declaration or any other relevant guidelines.

### Statistical analysis

Distribution of continuous data was inspected using histograms. Normally distributed data are presented as means and standard deviations (SD), while nonparametric data are presented as median, 25th percentile (P25), and 75th percentile (P75). Categorical data are presented as proportions. ALT levels as the dependent variable were tested using the chi-square test for comparison with categorical variables, as well as one-way analysis of variance (ANOVA) for comparison with continuous variables. Independent predictors of mildly and markedly increased ALT were analyzed using multivariate logistic regression. As there has been evidence of sex difference on the risk of elevated ALT in the adult population [17, 18], we examined the sex-age interaction effect on mildly and markedly increased ALT. The adjusted analyses were stratified by sex. The results are presented as odds ratios (ORs) with 99% confidence intervals (CIs). Statistical significance was defined as *p* < 0.01. Data were managed and analyzed using STATA® version 16 (StataCorp, College Station, Texas 77,845 USA).

## Results

### Characteristics

Of the 11,776 individuals included, 46.5% were female. The mean age of individuals was 11.0 ± 3.3 years. The proportions of class I, class II, and class III obesity were 56.3% (*n* = 6629), 28.4% (*n* = 3345), 15.3% (*n* = 1802), respectively. Sex and BMI SDS did not differ between individuals with and without ALT data. Yet, those with ALT data were 6 months older than those without ALT data (*p* < 0.001).

### Prevalence of mildly and markedly increased ALT

The prevalence of mildly increased ALT and markedly increased ALT were 37.9% (99% confidence interval [CI]: 36.7 to 39.0%) and 10.6% (99% CI: 9.9 to 11.3%), respectively. The median ALT level in the mildly increased ALT group was 30 U/L (P25 = 27 U/L, P75 = 36 U/L), and in the markedly increased ALT group; 66 U/L (P25 = 54 U/L, P75 = 90 U/L).

Compared with those with normal ALT, individuals with mildly and markedly increased ALT were older and had higher BMI SDS, all *p* < 0.001. Individuals with markedly increased ALT were more likely to be males (66.7% vs. 53.5% in normal ALT group, *p* < 0.001), whereas a lower proportion of males (49.8%) was found in mildly increased ALT group, *p* < 0.001. Characteristics of the individuals according to the ALT level are shown in Table 1.

**Table 1:** General characteristics of individuals in the obesity cohort

Variables	Total	Normal ALT	Mildly increased ALT	Markedly increased ALT	***P*** value^**1**^	***P*** value^**2**^	***P*** value^**3**^
			>URL to < 2 x URL	≥2 x URL			
	***n*** = 11,776	***n*** = 6070	***n*** = 4461	***n*** = 1245			
Males, n(%)	6300 (53.5)	3250 (53.5)	2220 (49.8)	830 (66.7)	**< 0.001**	**< 0.001**	**< 0.001**
Age (years), mean (SD)	11.0 (3.3)	10.7 (3.3)	11.1 (3.3)	12.5 (3.3)	**< 0.001**	**< 0.001**	**< 0.001**
5- < 8 years, n(%)	2574 (21.9)	1526 (25.1)	931 (20.9)	117 (9.4)			
8- < 11 years, n(%)	3427 (29.1)	1761 (29.0)	1365 (30.6)	301 (24.2)			
11- < 14 years, n(%)	3077 (26.1)	1603 (26.4)	1123 (25.2)	351 (28.2)			
14- < 18 years, n(%)	2698 (22.9)	1180 (19.4)	1042 (23.4)	476 (38.2)			
Degree of obesity							
Class I obesity, n(%)	6629 (56.3)	3815 (62.8)	2265 (50.8)	549 (44.1)	**< 0.001**	**< 0.001**	**< 0.001**
Class II obesity, n(%)	3345 (28.4)	1553 (25.6)	1386 (31.1)	406 (32.2)			
Class III obesity, n(%)	1802 (15.3)	702 (11.6)	810 (18.2)	290 (23.3)			

*Abbreviations*: *ALT* alanine aminotransferase, *SD* standard deviation, *URL* upper reference level

Differences between groups were assessed using the chi-square test or independent t-test, where appropriate

^1^ Comparison between mildly increased ALT and normal ALT group

^2^ Comparison between markedly increased ALT and normal ALT group

^3^ Comparison between markedly increased ALT and mildly increased ALT group

### Associations of mildly and markedly increased ALT with sex, age, and degree of obesity

Crude analyses indicated that age was associated with both mildly increased ALT (OR 1.04 for each year increase, 99% CI: 1.02 to 1.05, *p* < 0.001) and markedly increased ALT (OR: 1.19 for each year increase, 99% CI: 1.16 to 1.22, *p* < 0.001). On the other hand, male sex was positively associated with markedly increased ALT (OR: 1.73, 99% CI: 1.47 to 2.05, *p* < 0.001), yet negatively associated with mildly increased ALT (OR: 0.86, 99% CI: 0.78 to 0.95, *p* < 0.001). An interaction between sex and age was observed, *p* < 0.001.

**Fig. 2:**
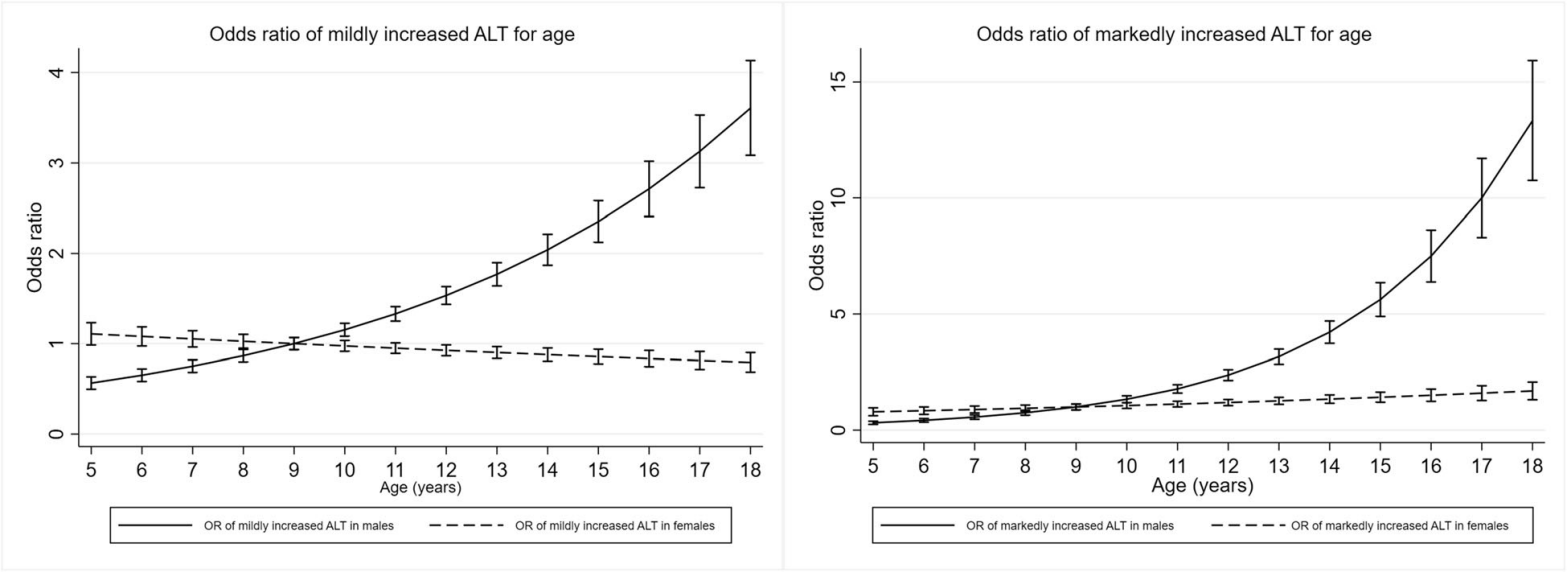
ORs with 99% CIs of mildly (**A**) and markedly (**B**) increased ALT for age, stratified by sex. Age of 9 years as reference. ORs were adjusted for BMI SDS. Solid line represents OR for males. Dashed line represents OR for females. I represents 99% CIs

Given the significant interaction between sex and age, adjusted ORs of mildly and markedly increased ALT for age were calculated for males and females, separately. With normal ALT group as the reference, the adjusted OR of markedly increased ALT in males was 1.34 (99% CI: 1.29 to 1.38, *p* < 0.001) vs 1.06 (99% CI: 1.02 to 1.10, *p* < 0.001) in females for each year of age increase. The adjusted OR of mildly increased ALT in males was 1.15 (99% CI: 1.13 to 1.18, *p* < 0.001) vs 0.97 (99% CI: 0.95 to 0.99, *p* = 0.002) in females for each year increase. The adjusted ORs of mildly and markedly increased ALT for age and degree of obesity, stratified by sex, are presented in Table 2. Graphs illustrating the associations of mildly and markedly increased ALT for age in males and females, separately, are shown in Fig. 2.

**Table 2:** ORs of mildly and markedly increased ALT^a^ for age and degree of obesity, stratified by sex

	OR (99% CI, ***P*** value)		OR (99% CI, ***P*** value)	
	Mildly increased ALT, ***n*** = 4461		Markedly increased ALT, ***n*** = 1245	
	Males	Females	Males	Females
Age (years)	1.15 (1.13–1.18, < 0.001)	0.97 (0.95–0.99, 0.002)	1.34 (1.29–1.38, < 0.001)	1.06 (1.02–1.10, < 0.001)
Degree of obesity				
Class I obesity	Ref	Ref	Ref	Ref
Class II obesity	1.52 (1.29–1.80, < 0.001)	1.57 (1.33–1.86 < < 0.001)	2.03 (1.60–2.58, < 0.001)	1.59 (1.15–2.20, < 0.001)
Class III obesity	2.27 (1.82–2.82), < 0.001)	2.17 (1.75–2.70, < 0.001)	3.73 (2.77–5.02, < 0.001)	3.57 (2.52–5.06, < 0.001)

*Abbreviations*: *ALT* alanine aminotransferase, *OR* odds ratio, *CI* confidence interval, *Ref* reference group

^a^ Compared to normal ALT group

ORs were adjusted for age and degree of obesity

The degree of obesity was associated with both mildly and markedly increased ALT in a dose-response manner. Compared with individuals with class I obesity, adjusted OR of markedly increased ALT was greater in those with class II obesity (OR: 1.82, 99% CI: 1.51 to 2.20, *p* < 0.001) and greatest in class III obesity (OR: 3.38, 99% CI 2.71 to 4.23, *p* < 0.001). Likewise, the adjusted OR of mildly increased ALT for class II obesity was 1.51 (99% CI: 1.35 to 1.70, *p* < 0.001), and for class III obesity was 2.17 (99% CI: 1.66 to 2.61, *p* < 0.001).

### Associations of mildly and markedly increased ALT with the metabolic parameters

Of 11,776 individuals included in the study, 9223 individuals had fasting glucose and lipid profile data. Individuals with mildly and markedly increased ALT had higher proportions of impaired fasting glucose, high total cholesterol, high LDL, low HDL, and hypertriglyceridemia compared with normal ALT group, *p* < 0.001 for all analyses (Table 3).

**Table 3:** Metabolic characteristics of individuals in the obesity cohort

	All individuals	Normal ALT	Mildly increased ALT	Markedly increased ALT	***P*** value^**1**^	***P*** value^**2**^	***P*** value^**3**^
	(***n*** = 9223)	(***n*** = 4789)	(***n*** = 3527)	(***n*** = 907)			
Impaired fasting glucose	1897 (20.6)	916 (19.1)	718 (20.4)	263 (29.0)	**0.163**	**< 0.001**	**< 0.001**
LDL-C							
Borderline-high	1902 (20.6)	943 (19.7)	758 (21.5)	201 (22.2)	**< 0.001**	**< 0.001**	0.015
High	1517 (16.5)	671 (14.0)	645 (18.3)	201 (22.2)			
Total cholesterol							
Borderline-high	2760 (29.9)	1396 (29.2)	1094 (31.0)	270 (29.8)	**< 0.001**	**< 0.001**	0.029
High	1183 (12.8)	534 (11.2)	491 (13.9)	158 (17.4)			
Low HDL-C	1334 (14.5)	554 (11.6)	557 (15.8)	223 (24.6)			
Triglycerides							
Borderline-high	2126 (23.1)	1094 (22.8)	804 (22.8)	228 (25.1)	**< 0.001**	**< 0.001**	**< 0.001**
High	2445 (26.5)	956 (20.0)	1105 (31.3)	384 (42.3)			

*Abbreviations*: *ALT* alanine aminotransferase, *TC* total cholesterol, *LDL-C* low-density lipoprotein cholesterol, *HDL-C* high-density lipoprotein cholesterol, *OR* odds ratio, *CI* confidence interval

Differences between groups were assessed using the chi-square test

^1^ Comparison between mildly increased ALT and normal ALT group

^2^ Comparison between markedly increased ALT and normal ALT group

^3^ Comparison between markedly increased ALT and mildly increased ALT group

In a subset of individuals, 5262 (44.7%) had HOMA data available. Individuals in markedly increased ALT group (*n* = 560) had higher HOMA (median = 4.0, P25 = 1.4, P75 = 7.8) compared to those in mildly increased ALT group (*n* = 2027, median HOMA = 3.1, P25 = 1.4, P75 = 5.3) and those in normal ALT group (*n* = 2655, median HOMA = 2.7, P25 = 1.3, P75 = 4.3), *p* < 0.001 for comparison of markedly increased ALT vs normal ALT and markedly increased ALT vs mildly increased ALT group.

In multiple logistic regressions adjusted for sex, age, and degree of obesity, the following factors were associated with mildly increased ALT: borderline-high LDL (OR: 1.20, 99% CI: 1.04 to 1.39, *p* = 0.001), high LDL (OR: 1.41, 99% CI: 1.20 to 1.65, *p* < 0.001), borderline-high total cholesterol (OR: 1.17, 99% CI: 1.02 to 1.33, *p* = 0.002), high total cholesterol (OR: 1.37, 99% CI: 1.14 to 1.64, *p* < 0.001), low HDL (OR: 1.25, 99% CI: 1.05 to 1.49, *p* = 0.001), borderline-high triglycerides (OR: 1.16, 99% CI 1.00 to 1.34, *p* = 0.008), high triglycerides (OR: 1.78, 99% CI 1.54 to 2.05, *p* < 0.001), and HOMA (OR: 1.06, 99% CI 1.04 to 1.09, *p* < 0.001).

All metabolic parameters associated with mildly increased ALT had stronger associations with markedly increased ALT. In addition, impaired fasting glucose was associated with markedly increased ALT (OR: 1.62, 99% CI: 1.10 to 2.40, *p* = 0.001), but not with mildly increased ALT (OR: 1.21, 99% CI 0.91 to 1.62, *p* = 0.085). Adjusted ORs of mildly and markedly increased ALT for the metabolic parameters are shown in Table 4.

**Table 4:** ORs of mildly and markedly increased ALT^a^ for impaired fasting glucose and lipid profile

	Mildly increased ALT, ***n*** = 3527		Markedly increased ALT, ***n*** = 907	
	OR (99% CI)	***P*** value	OR (99% CI)	***P*** value
Impaired fasting glucose	1.21 (0.91, 1.62)	0.085	1.62 (1.10, 2.40)	**0.001**
LDL				
Acceptable	Ref		Ref	
Borderline-high	1.20 (1.04, 1.39)	**0.001**	1.34 (1.06, 1.71)	**0.002**
High	1.41 (1.20, 1.65)	**< 0.001**	1.77 (1.38, 2.27)	**< 0.001**
Total cholesterol				
Acceptable	Ref		Ref	
Borderline-high	1.17 (1.02, 1.33)	**0.002**	1.20 (0.97, 1.50)	**0.028**
High	1.37 (1.14, 1.64)	**< 0.001**	1.80 (1.37, 2.36)	**< 0.001**
HDL				
Acceptable	Ref		Ref	
Low	1.25 (1.05, 1.49)	**0.001**	1.69 (1.33, 2.15)	**< 0.001**
Triglycerides				
Acceptable	Ref		Ref	
Borderline-high	1.16 (1.00, 1.34)	**0.008**	1.65 (1.28, 2.11)	**< 0.001**
High	1.78 (1.54, 2.05)	**< 0.001**	3.06 (2.44, 3.84)	**< 0.001**
HOMA^b^	1.06 (1.04–1.09)	**< 0.001**	1.13 (1.09–1.17)	**< 0.001**

*Abbreviations*: *ALT* alanine aminotransferase, *TC* total cholesterol, *LDL-C* low-density lipoprotein cholesterol, *HDL-C* high-density lipoprotein cholesterol, *HOMA* homeostasis model assessment, *OR* odds ratio, *CI* confidence interval, *Ref* reference group

All ORs were adjusted for age, sex, and the degree of obesity

^a^ Compared to individuals with normal ALT

^b^ Analyses were based on 5262 individuals who had HOMA data (individuals with normal ALT, *n* = 2655; mildly increased ALT, *n* = 2047; markedly increased ALT, *n* = 560)

## Discussion

Results from this cohort of children and adolescents with obesity in Sweden, derived from 11,776 patients, presented a prevalence of mildly increased ALT of 37.9% and markedly increased ALT of 10.6%. Moreover, the results demonstrated evidence of sex differences in the associations of age with both mildly and markedly increased ALT. We also found dose-response associations of degree of obesity with both mildly and markedly ALT. Regarding metabolic parameters, high triglycerides, high LDL, high total cholesterol, and low HDL were associated with both mildly and markedly increased ALT. However, impaired fasting glucose was only associated with markedly increased ALT, but not with mildly increased ALT.

The prevalence of mildly and markedly increased ALT in this study was similar to a population of children and adolescents with overweight and obesity in European German-speaking countries [20], where the prevalence of mildly and markedly increased ALT were 35.7 and 13.5%, respectively. When considering mildly and markedly increased ALT as an entity, a lower prevalence of increased ALT (16.8%) was reported in an epidemiological study of children and adolescents with obesity in Israel [33]. Compared to our finding, this lower prevalence of increased ALT may be due to hereditary and ethnic differences, as well as different dietary patterns, as they are known to influence NAFLD risk [13, 34, 35].

Although ALT is commonly used as a proxy for NAFLD, liver biopsy remains the gold standard in the diagnosis of NAFLD [14, 24]. Compared with the prevalence of markedly increased ALT of 10.6% in our study, an autopsy-based study in the US reported a higher prevalence of NAFLD based on liver biopsy, which was 38% in children and adolescents with obesity [13]. However, since the sample in the US study was deceased individuals whose causes of death were mostly due to accidents, homicide, and suicide, the finding in the US study might not represent general pediatric population with obesity. Although ALT is an important indicator for childhood NAFLD, it is not enough as a single marker. Thus, a combination of clinical, biochemical, biomarkers and non-invasive imaging tests may replace liver biopsy in the future. On the other hand, liver function tests and non-invasive measurements, such as transient elastography together with controlled attenuation parameter, may improve the accuracy of non-invasive diagnosis of those children with obesity and NAFLD-fibrosis who should undergo a liver biopsy. The indication of liver biopsy is not only to diagnose NAFLD per se, but to rule out steatohepatitis which is more likely to progress to advanced fibrosis and cirrhosis [10].

Although several studies have investigated the association of elevated ALT with age and sex among children and adolescents with obesity [15, 16, 33], previous studies did not take sex-age interaction into account in the analyses. The present study confirms that both sex and age are independently associated with elevated ALT. In addition, the present study provides evidence of sex difference in the associations of age with both mildly and markedly increased ALT. The odds of markedly increased ALT in adolescent males with obesity was increased dramatically over the years, while the corresponding pattern in adolescent females with obesity was minuscule. Considering increased ALT as a marker for NAFLD, the sex-age interaction in NAFLD has been previously shown in adult population [17, 18, 36]; NAFLD is more common in males during young and middle adulthood, yet after the ages of 50 the NAFLD prevalence is higher in females. Although the mechanism of the age-sex interaction in NAFLD is still uncertain, a possible explanation is that estrogen may protect the liver from injuries and steatosis [17, 36]. Moreover, mice models showed that, in high-fat environment, the innate immune cells from males are activated and thereby promote liver inflammation, while macrophage in females produce higher anti-inflammatory lipid mediators. The different inflammatory responses between male and female mice appeared to be due to sex hormone differences as the anti-inflammatory response in female mice was lost with ovariectomy [17]. Furthermore, female is suggested to be more responsive to browning of white adipose tissue which would increase free fatty acid metabolism and thus may reduce the risk for hepatotoxicity [3]. Another possible explanation for the sex-age interaction in NAFLD among children and adolescent with obesity is that insulin resistance, which play important role in NAFLD pathogenesis, is more prevalent in male adolescents with obesity compared to female adolescents with obesity [37]. Understanding the sex-age interaction in pediatric NAFLD would support future pediatric NAFLD screening guidelines based on age and sex.

In accordance with other studies [15, 16], the present study found a dose-response association between the degree of obesity and increased ALT among children and adolescents with obesity. Moreover, we have been able to show that the dose-response association is stronger in markedly increased ALT compared to mildly increased ALT. Compared to individuals with class I obesity, those with class III obesity had 2.2-fold higher odds of having mildly increased ALT and 3.7-fold higher odds of having markedly increased ALT. There are some probable mechanisms of the positive association between the degree of obesity and increased ALT. Firstly, a higher degree of obesity contributes to higher ectopic fat accumulation in the liver leading to liver injury [38]. Secondly, a higher degree of obesity is associated with more elevated circulating inflammatory cytokines [39]. Furthermore, the degree of obesity is correlated with insulin resistance [40], and insulin resistance is linked with the inflammatory response [41].

In line with other studies in children and adolescents with obesity [20, 21], the present study demonstrated the association of markedly increased ALT with impaired fasting glucose, independent of the degree of obesity. This confirms that insulin resistance plays an important role in the pathogenesis of pediatric NAFLD, as explained by the “multiple-hit model”; considered as the first hit, insulin resistance and obesity are the main factors inducing the first lipid accumulation in the liver [42]. Furthermore, systemic and hepatic insulin resistance also contributed to oxidative damage, leading to hepatocellular death and progression of liver steatosis to non-alcoholic steatohepatitis, fibrosis and cirrhosis [43].

While pediatric NAFLD has been positively associated with atherogenic lipid components in the general population [44–47], study on this association in children and adolescents with obesity demonstrated diverging results [15, 21, 23, 48]. A hospital-based study indicated association between NAFLD and dyslipidemia [48], while a large population-based study did not detect such association [15]. Other hospital-based studies found association of NAFLD with triglycerides [21, 23] and total cholesterol [21], but not with other lipid components. These diverging results may be due to various cut-offs values for ALT and dyslipidemia components used in those studies. The present study has been able to divide ALT and each lipid components into varying degrees. Our results indicated stepwise patterns of the association between each atherogenic lipid component with increased ALT - the higher the total cholesterol, LDL, and triglyceride levels, the higher the odds of having mildly increased ALT. When the outcome was markedly increased ALT, its association with those lipid components were stronger. Our results are consistent with recent findings from a cohort of children and adolescents with obesity in Israel [33]. However, the associations in the Israeli study were not adjusted, while our results were adjusted for the degree of obesity, age, and sex.

Compared with other lipid components, the present study showed that triglyceride levels had the strongest association with both mildly increased ALT and markedly increased ALT. While the present study used ALT as a surrogate marker for NAFLD, other studies using ultrasound [21, 23] and liver biopsy [22] to diagnose NAFLD also indicated that high level of triglycerides was associated with pediatric NAFLD in obese population. Further, a study in a cohort of pediatric patients with biopsy-proven NAFLD suggested that a high level of triglycerides was a risk factor for developing non-alcoholic steatohepatitis [49].

### Limitations and strengths of the study

The gold standard of NAFLD diagnosis is liver biopsy. Yet, it is not possible to perform liver biopsy in a large epidemiological study due to its invasiveness. We have used ALT as a marker of suspected NAFLD, and hence few considerations should be considered in interpreting the results. Firstly, other conditions than liver disease, e.g., viral infections, muscle disease, and drug-induced liver injury [29], may affect ALT levels in children and adolescents. Especially in children younger than 5 years of age, increased transaminases are likely to have other causes [9]. In addition, among children aged 3–10 years, other liver diseases (e.g., Wilson’s disease, celiac disease, viral hepatitis) are usually more probable than NAFLD [9]. Secondly, although ALT have been known to have high sensitivity in identifying NAFLD in children with overweight and obesity [8], 17–22% cases of pediatric liver steatosis had normal ALT level [28, 50]. Moreover, degree of ALT elevation does not correlate with steatosis severity [51]. Individuals in the advanced stage of NAFLD, i.e., fibrosis and cirrhosis, may have normal values of ALT. Misclassification bias therefore might occur in the present study. Thirdly, as ALT was measured in a single time point in the present study, we might miss the within-individual biological variation. Yet, the National Health and Nutrition Examination Survey (NHANES) involving adolescents with obesity in US found that diurnal variation did not affect the prevalence of elevated ALT [4]. Another study found that children and adolescents with ALT > 2 times URL tend to have persistently elevated ALT [29]. In the present study, we did not use AST as concomitant marker to ALT, as it has not been evaluated in pediatric obesity-related NAFLD.

We chose the first recorded ALT obtained in obesity treatment. Nevertheless, obesity duration before the initiation of treatment remains unknown since the BORIS register only provides data during treatment and not historical measures. In addition, we had no information on ethnicity, whereas some studies indicated association of pediatric NAFLD with ethnicity [13, 34]. Hence, the generalizability of the results in the present study to other populations may be limited. Nevertheless, approximately one-third of individuals in the BORIS register have a non-Nordic background [6].

A primary strength of the study is the data taken from a national clinical register. This strengthens the external validity of the study. Moreover, the large sample size made it possible to categorize the individuals into varying degrees of increased ALT, degree of obesity severity, and dyslipidemia to show the patterns of association between increased ALT and degree of obesity as well as metabolic parameters.

## Conclusions

Results from this large cohort of children and adolescents with obesity show that male sex, increasing age, severity of obesity, low HDL, increased level of triglycerides, LDL cholesterol, total cholesterol, and HOMA are independently associated with mildly and markedly increased ALT. Moreover, there is an interaction effect between age and sex which modulates the risk of increased ALT. Regular NAFLD screening in children and adolescents with obesity who have highest risk of increased ALT is recommended to promote early detection and prompt treatment of pediatric NAFLD.

## Data Availability

Data cannot be shared publicly because of third-party data. Data are available from Statistics Sweden (contact via information@scb.se), the Swedish National Board of Health and Welfare (contact via registerservice@socialstyrelsen.se), and the Swedish Childhood Obesity treatment Register (contact via http://www.e-boris.se/kontaktuppgifter/), for researchers who meet the criteria for access to confidential data given by the Ethics Committee (contact via registrator@etikprovning.se).

## Abbreviations

ALT: Alanine aminotransferase
CI: Confidence interval
HDL-C: High-density lipoprotein cholesterol
HOMA: Homeostasis model assessment
LDL-C: Low-density lipoprotein cholesterol
NAFLD: Non-alcoholic fatty liver disease
OR: Odds ratio
P25: 25th percentile
P75: 75th percentile
Ref: Reference group
SD: Standard deviation
TC: Total cholesterol
URL: Upper reference level
